# Seroepidemiological Survey for *Coxiella burnetii* Antibodies and Associated Risk Factors in Dutch Livestock Veterinarians

**DOI:** 10.1371/journal.pone.0054021

**Published:** 2013-01-16

**Authors:** René Van den Brom, Barbara Schimmer, Peter M. Schneeberger, Wim A. Swart, Wim van der Hoek, Piet Vellema

**Affiliations:** 1 Department of Small Ruminant Health, Animal Health Service, Deventer, The Netherlands; 2 Centre for Infectious Disease Control, National Institute for Public Health and the Environment, Bilthoven, The Netherlands; 3 Department of Medical Microbiology and Infection Control, Jeroen Bosch Hospital, ‘s-Hertogenbosch, The Netherlands; 4 Department of Diagnostics, Research and Epidemiology, Animal Health Service, Deventer, The Netherlands; University of Iowa, United States of America

## Abstract

Since 2007, Q fever has become a major public health problem in the Netherlands and goats were the most likely source of the human outbreaks in 2007, 2008 and 2009. Little was known about the consequences of these outbreaks for those professional care providers directly involved. The aim of this survey was to estimate the seroprevalence of antibodies against *C. burnetii* among Dutch livestock veterinarians and to determine possible risk factors. Single blood samples from 189 veterinarians, including veterinary students in their final year, were collected at a veterinary conference and a questionnaire was filled in by each participant. The blood samples were screened for IgG antibodies against phase I and phase II antigen of *C. burnetii* using an indirect immunofluorescent assay, and for IgM antibodies using an ELISA. Antibodies against *C. burnetii* were detected in 123 (65.1%) out of 189 veterinarians. Independent risk factors associated with seropositivity were number of hours with animal contact per week, number of years graduated as veterinarian, rural or sub urban living area, being a practicing veterinarian, and occupational contact with swine. Livestock veterinarians should be aware of this risk to acquire an infection with *C. burnetii*. Physicians should consider potential infection with *C. burnetii* when treating occupational risk groups, bearing in mind that the burden of disease among veterinarians remains uncertain. Vaccination of occupational risk groups should be debated.

## Introduction

Q fever is a zoonotic disease caused by the obligate intracellular bacterium, *Coxiella burnetii*, and ruminants are considered to be the primary source of infection for humans. In cattle, the disease is mainly asymptomatic [1], but in sheep and goats the main symptom is abortion, stillbirth and retention of foetal membranes [2]–[6]. The bacterium is shed in urine, milk, faeces and birth products of infected animals. The main route of transmission of the bacterium to humans is by aerosols [4], [7], [8].

Until 2007, about 20 Q fever cases were reported in the Netherlands annually [9]. In that year, Q fever became a major public health problem in the Netherlands with 168, 1000 and 2,357 human cases notified in 2007, 2008 and 2009, respectively [10]. These unprecedented annual outbreaks are largely explained by exposure of the general population living in the surroundings of infected dairy goat farms to airborne contaminated dust particles. Only 5% of the notified Q fever patients in the Netherlands report an occupation in agriculture, transporting or handling animal products, or animal care [11]. However, since its first description in abattoir workers in Australia in 1935 [12], Q fever has been considered primarily an occupational zoonotic disease for abattoir workers, sheep shearers, livestock farmers, and especially veterinarians because of their direct contact with potentially infected animals [13]–[19].

The aim of this survey was to estimate the seroprevalence of antibodies against *C. burnetii* among Dutch livestock veterinarians and to determine possible risk factors.

## Materials and Methods

### Human Population and Data Collection

In November 2009, professional laboratory assistants collected a single blood sample from Dutch livestock veterinarians and final-year veterinary students attending a veterinary conference.

Each participant filled in a self-administered questionnaire to obtain epidemiological and clinical information. The questionnaire existed of three parts, and took approximately fifteen minutes to complete. The first part focused on demographic data and included age, gender, and residence in urban, sub urban or rural area. The second part consisted of occupation-related questions regarding work location, type of veterinary occupation, years in veterinary practice, contact with livestock and livestock farms, contact with animal related products as straw, hay, soil, birth products and urine and faeces, contact with aborted animals, use of personnel protective equipment, work related wounds and accidental vaccine exposure. The third part consisted of non-occupation related questions regarding possession of animals in the last five years, consumption of raw dairy products, outdoor activities and health conditions, including smoking, tick bites during the last five years and a known history of a clinical Q fever infection, pregnancy and abortion.

This study was approved by the Medical Ethical Committee of the University Medical Centre Utrecht, Utrecht, the Netherlands (reference number 09–322). All participants received a book to express appreciation for their cooperation.

### Laboratory Methods

A serum sample from each participant was tested for the presence of IgG antibodies against C. burnetii using a Q fever indirect immunofluorescent assay (IFA; Focus Diagnostics, Cypress, CA), according to the manufacturer’s protocol. Sera were screened for phase I and phase II IgG using a cut-off of 1∶32. Samples with both IgG phase I and II titres of ≥1∶32 were considered to be positive, while solitary IgG phase II samples were scored positive if they had a single titre of ≥1∶512.

All samples were also screened for IgM using an ELISA (Focus Diagnostics), according to the manufacturer’s protocol, and positive samples were confirmed with IFA. Samples with a titre of ≥1∶32, both for IgM phase I and II, were considered to be positive, indicating a possibly recent infection.

Within the group of participants with a past infection, a distinction was made between serological profiles considered not likely to be compatible with a chronic infection, and serological profiles which could indicate a chronic infection. Serum samples from participants with a possibly chronic Q fever infection, having an IgG phase I titre ≥1∶1024, were additionally analysed by performing a *C. burnetii* PCR.

### Statistical Data Analysis

All individual laboratory results were merged with the self-administered questionnaires. Statistical analysis was carried out using STATA 11. The Chi square test and the two-sided proportion-test were used to estimate univariate associations between exposures and seropositivity. Analyses were carried out to calculate odds ratio’s with 95% confidence intervals. The odds ratio (OR) was defined, in this context as the odds of a given exposure among veterinarians seropositive for *C. burnetii* divided by the odds of exposure among seronegative veterinarians. Veterinarians who did not completely fill in the questionnaire were excluded for the analysis of that particular question.

For the multivariable logistic regression, initially all variables with (2-sided) p<0.20 and with sufficient numbers (>10) were selected. To avoid multicollinearity, from groups of variables that had a correlation of more than 0.50 with each other, only one, the most plausible biological variable, was left in the multivariable analysis.

Stepwise backward logistic regression was carried out, starting with all data and excluding stepwise each variable that had a p-value of >0.05. All remaining variables were considered to be risk or protective factors.

## Results

### Descriptive Results

A total of 189 participants, being more than 90% of the attendants, completed the questionnaire and provided a blood sample during the conference. The median age of the participants was 44 years (interquartile range, 34–52 years). Of the participants, 130 (68.8%) were male and 59 (31.2%) were female (Table 1). One hundred and twelve of the participants worked as a livestock practitioner, 20 were non-practicing, 37 worked as livestock veterinarian at a veterinary institute (Utrecht University (UU) or Animal Health Service (GD)) and 20 were livestock veterinary students in their final year. A total of 108 (57.1%) of the participants had contact with livestock for more than 50% of working hours in their current job.

**Table 1:** Results of univariable analysis of risk factors for presence of antibodies against *Coxiella burnetii*.

	Participants							
	Seropositive#		Seronegative					
	No.	%	No.	%	Odds Ratio	95% confidence interval		P
**Gender**								
Female	24	40.7	35	59.3	1.0	.	.	.
Male	99	76.2	31	23.8	4.7	2.3	9.4	<0.001
**Age**								
< = 32 year	19	40.4	28	59.6	1.0	.		.
33–44 year	35	71.4	14	28.6	3.7	1.6	8.6	0.003
45–52 year	37	75.5	12	24.5	4.5	1.9	10.9	0.001
53–65 year	32	72.7	12	27.3	3.9	1.6	9.5	0.002
**Living region**								
Urban	8	30.8	18	69.2	1.0	.	.	.
Sub-urban	21	56.8	16	43.2	3.0	1.0	8.5	0.037
Rural	94	74.6	32	25.4	6.6	2.6	16.7	<0.001
**Veterinarian (years)**								
Veterinarian (< = 2)	13	27.7	34	72.3	1.0			
veterinarian (3–13)	36	70.6	15	29.4	6.3	2.6	15.1	<0.001
veterinarian (14–21)	33	75.0	11	25.0	7.9	3.1	20.0	<0.001
veterinarian (> = 22)	40	87.0	6	13.0	17.4	6.00	50.8	<0.001
**Animal contact (hours/week)**								
<10 hours	9	24.3	28	75.7	1.0			
10–19 hours	25	55.6	20	44.4	3.9	1.5	10.1	0.005
20–29 hours	42	80.8	10	19.2	13.1	4.7	36.2	<0.001
> = 30 hours	43	89.6	5	10.4	26.8	8.1	88.2	<0.001
**Work category**								
Others	23	30.7	52	69.3	1.0			
Practicing	100	87.7	14	12.3	16.2	7.7	34.0	<0.001
**Contact with cows**								
No	11	31.4	24	68.6	1.0	.	.	.
Yes	112	72.7	42	27.3	5.8	2.6	12.9	<0.001
**Contact with swine**								
No	80	61.5	50	38.5	1.0	.		.
Yes	43	72.9	16	27.1	1.7	0.9	3.3	0.131
**Contact with birth products of ruminants**								
No	16	33.3	32	66.7	1.0	.	.	.
Yes	107	75.9	34	24.1	6.3	3.1	12.9	<0.001
**Contact with birth products of pets**								
No	101	61.2	64	38.8	1.0	.	.	.
Yes	22	91.7	2	8.3	7.0	1.5	31.9	0.004
**Practice on cow farm with abortion**								
No	32	43.8	41	56.2	1.0	.	.	.
Yes	91	78.4	25	21.6	4.7	2.4	9.3	<0.001

# Sera were screened for phase I and phase II IgG using a cut-off of 1∶32. Samples with both IgG phase I and II ≥1∶32 were considered to be positive, while solitary IgG phase II samples were scored positive if they had a single titre of ≥1∶512 (Focus Diagnostics, Cypress, CA).

The overall seroprevalence was 65.1% (n = 189). In livestock veterinarians the seroprevalence was 69.2% (n = 169). The seroprevalence in livestock veterinary students was 30.0% (n = 20). Among the group of 169 livestock veterinarians the seroprevalence was 87.5% in practicing livestock veterinarians (n = 112), 45.0% in non-practicing livestock veterinarians (n = 20) and 27.0% in livestock veterinarians working at a veterinary institute (n = 37). IgG antibody titers against *C. burnetii* measured for both phase I and II ranged from 1∶32 to 1∶2048. Seven out of nine participants with a positive IgM ELISA result were confirmed with IFA, suggesting a recent infection. Four of those seven IFA positive study participants were livestock veterinary students. The other three were practicing livestock veterinarians. Seven participants with an IgG phase I titre ≥1∶1024, a possible indication of a chronic Q fever infection, were followed up by performing a *C. burnetii* PCR on a blood sample, and in all cases PCR results were negative. Additionally, participants with an IgG phase I titre ≥1∶512 are offered to participate in a follow-up study and are advised to be controlled for risk factors of a chronic Q fever infection.

### Univariable Analysis

Participants who were seropositive were likely to be male over the age of 32 years (Table 1). Participants living in rural or suburban areas were significantly more often seropositive than participants living in an urban area. Occupational risk factors in univariable analysis were: graduated as a veterinarian more than two years ago; more than 10 hours of animal contact per week; practicing as livestock veterinarian; and working with cattle, horses, dogs and cats. Participants with frequent contact with animal products, like straw, hay, roughage, raw milk, birth products of ruminants as well as of pets, urine of ruminants, practicing on cattle farms with abortion, and one or more contacts on farms with abortion problems in the last five years, were significantly more often seropositive. Accidental needle injections and cutting incidents were also found to be associated with seropositivity. Non-occupational activities like cycling and shopping were associated with seronegativity. In contrast, gardening and having dogs and (pet) birds were found to be associated with seropositivity. Consumption of dairy products, health conditions like smoking behaviour, and not wearing protective clothes during work were not found to be a significant univariate risk factor. The number of participants primarily working with sheep and goats, with a history of a clinical Q fever infection, or with pregnancy and abortion was too small for statistical analysis.

### Multivariable Analysis

Variables with a p-value <0.20 in the univariable analysis were used as input for the multivariable analysis. The number of years as a veterinarian was highly correlated with age and gender; the latter two were left out of the analysis. Working category and contacts with ruminants were very highly correlated to contact with hay/straw, roughage, raw milk, birth products of ruminants and with urine of ruminants; the latter 5 were left out of the analysis.

In this group of livestock veterinarians, risk factors for *C. burnetii* seropositivity in the multivariable analysis (Table 2) were: number of hours with animal contact per week, number of years graduated as veterinarian, living in a rural (OR, 17.9 (95% CI: 3.6–88.1)) or semi urban area (OR, 11.9 (95% CI: 2.1–68.5)), working as practicing livestock veterinarian (OR, 15.8 (95% CI: 2.9–87.2)), and occupational contact with swine (OR, 3.4 (95% CI: 1.1–10.2)).

**Table 2:** Final multivariable model for risk factors associated with presence of antibodies against *Coxiella burnetii* in 189 veterinarians.

Variable	Category	No.	OR	[95% CI]		P
Animal contacts	<10 hours	37	1.0			
(hours/week)	10–19 hours	45	12.0	2.5	57.1	0.002
	20–29 hours	52	1.2	0.2	7.6	0.869
	> = 30 hours	48	16.0	1.8	141.8	0.013
Veterinarian (years)	< = 2	47	1.0			
	3–13	51	17.5	4.0	77.4	<0.001
	14–21	44	26.5	4.8	145.9	<0.001
	> = 22	46	58.1	10.3	328.0	<0.001
Living region	Urban	26	1.0			
	Sub-urban	37	11.9	2.1	68.5	0.005
	Rural	126	17.9	3.6	88.1	<0.001
Work category	Others	75	1.0			
	Practicing	114	15.8	2.9	87.2	0.002
Contact with swine	No	130	1.0			
	Yes	59	3.4	1.1	10.2	0.029

## Discussion

In this cross-sectional study, an overall *C. burnetii* seroprevalence of 65.1% among Dutch livestock veterinarians was found. The number of hours with animal contact per week, the number of years the participants were graduated and practicing as a veterinarian, were the main independent risk factors in this study. These risk factors suggest a high dose-effect relation for seropositivity in Dutch livestock veterinarians. In 1984, 84% of 222 Dutch livestock veterinarians were seropositive for IgG antibodies against *C. burnetii* [17]. The use of a different laboratory test and cut-offs, differences in study population and different infection rates of livestock over time could be possible explanations for other seroprevalence estimates.

Dutch livestock veterinarians have a high risk of getting *C. burnetii* seropositive because of intensive contact with potentially infected livestock, and the immune system can be boosted frequently because of a high prevalence in Dutch livestock [20], [21]. Contact with swine was found to be an independent risk factor, but the group of veterinarians involved was also exposed to cattle. Further, the main geographical areas where pigs are kept in the Netherlands corresponds with the high-incidence areas where the human Q fever epidemic related to dairy goats was situated and where high seroprevalences were found in the rural population. On the other hand, treatment of swine has previously been described as a risk factor for seropositivity for veterinarians [19]. The natural susceptibility of swine to *C. burnetii* was demonstrated during a Q fever epidemic in Uruguay. A seroprevalence of 21.4% was measured in 391 healthy slaughter pigs [22]. No information about Q fever prevalences in swine in the Netherlands is available.

In this survey, 20 veterinary students participated, and the seroprevalence was 30%. In a survey in Spain, a seroprevalence of 11% among veterinary students was found. First course students showed a significant lower seroprevalence. Multiple risk factors were associated with *C. burnetii*: study course, contact with live animals especially ruminants and contact with persons working with animals [18]. A large serological survey (n = 674) was already carried out in the Netherlands in 2006. At that time 18.7% of the veterinary students were seropositive. Students in their final year with the livestock study direction had a seroprevalence of 37.3%. The main risk factors were a study direction focusing on large animals, advanced year of study, having had a zoonosis during study and having ever lived on a farm with ruminants [23]. To detect possible recent exposure to *C. burnetii*, testing was also performed by ELISA IgM, and it is not remarkable that four out of seven possible recent infections occurred in veterinary livestock students, indicating this group is susceptible for the infection during the practical rotations during their study. The lower prevalence in veterinary students, an indication for recent infection in seven of whom four were students, and the main risk factors we found, are another indication for a high dose-effect relation for seropositivity.

Our study clearly indicates that livestock veterinarians are an occupational risk group. The prevalence found in this study was much higher than described in several international sero-epidemical studies among livestock veterinarians [13]–[15], [18], [19], [24], [25], with the exception of a small survey among 12 veterinarians in southern Italy, which revealed a seroprevalence of 100% [16]. In other studies, contact with livestock is described as an important risk factor for seropositivity [14], [19], [24], and exposure to goats was the most important risk factor associated with *C. burnetii* infection in Southern Taiwan [14]. Treatment of cattle, swine or wildlife were main risk factors associated with *C. burnetii* seropositivity in US veterinarians [19]. In Slovakia and Nova Scotia, professional orientation and regular contact with farm animals and pets [24], and exposure to sheep placentas [15] were described as important risk factors, respectively. In contrast, in Japan, no significant correlation was found between years of occupational experience and *C. burnetii* seropositivity [13].

The final independent risk factor was living in a rural or sub-urban area. Participants living in these areas were significantly more often seropositive than participants living in an urban area. Rural and sub-urban living areas have been described before as a risk factor [26]–[30], although urban outbreaks also have been described, but could mostly be related to exposure to animals or animal products [31]–[33]. In the Netherlands, livestock farms are mainly situated in rural or sub-urban areas. The knowledge that ruminants are the main reservoir for *C. burnetii* [1], [34] and the fact that *C. burnetii* can easily be spread by aerosols [4], [7], [8], presumably explains why living in rural or sub-urban area is a risk factor for seropositivity.

In the univariable analysis, age and gender were risk factors for seropositivity. Nevertheless, both were left out of the multivariable analysis because they were highly correlated with the number of years participants were graduated as veterinarian. The higher incidence in males than in females has been reported in several sero-epidemical studies among veterinarians, but without a clear explanation [15], [17]–[19]. Also a Spanish study among veterinary students revealed that male students in the fifth study year had a significantly higher risk to be seropositive than female students [18]. A higher clinical incidence in males and persons aged between 40–60 years in the Dutch population has been described during the Q fever outbreaks between 2007–2010 [11]. Age above 46 years, was also previously described as a risk factor for seropositivity in veterinarians [19].

To differentiate in the group of practicing veterinarians, all analyses were repeated in the multivariable analysis for the subset of practicing veterinarians only, mainly working with cattle, swine and poultry, or individual housed animals. The analysis on the subset of practicing veterinarians did not result in additional significant results (data are not shown), and was less robust than the multivariable analysis based on the full data set.

In conclusion, Dutch livestock veterinarians are an occupational risk group with increased risk for *C. burnetii* infection presumably because of their direct contact with infected livestock. Dutch livestock veterinarians should be aware of this risk and be extra alert regarding symptoms of Q fever. Most of the infections are not notified, as they remain asymptomatic or result in only mild flu-like symptoms. Serious infections leading to pneumonia or hepatitis may occur. A *C. burnetii* infection can cause serious complications during pregnancy and in those with underlying disease, therefore these groups should be monitored properly. Vaccination of occupational groups at risk is common in Australia [35], [36]. In the Netherlands, vaccination has been made available in the first half of 2011, but only for specific risk groups, as those patients with heart valve and vascular disorders. During the community Q fever outbreaks between 2007 and 2009 in the Netherlands, few patients reported occupational exposure [11]. Most veterinarians are not eligible for vaccination because the presence of antibodies is an absolute contraindication for administering the currently available Australian vaccine. However, vaccination could be considered for seronegative veterinary students at the beginning of their study [35]. Routine serological follow-up is useful as well as basic safety rules, like hygiene measures and the use of protection clothes [18], [19], [24], [37], although in this study disregard of protective measures was not found to be an independent risk factor. Occupational exposure to several zoonotic diseases makes basic safety rules useful for protecting the livestock veterinarian.
